# Revisiting additive and dominance effects

**DOI:** 10.1093/g3journal/jkag139

**Published:** 2026-06-22

**Authors:** Jeffrey B Endelman, Julie C Dawson

**Affiliations:** Department of Plant & Agroecosystem Sciences, University of Wisconsin-Madison, Madison, WI 53706, United States; Department of Plant & Agroecosystem Sciences, University of Wisconsin-Madison, Madison, WI 53706, United States

**Keywords:** genomic prediction, orthogonal parameterization, inbreeding coefficient

## Abstract

The concept of additive and dominance effects is central to quantitative genetics, having been first proposed by R.A. Fisher in 1918. Despite this long history, confusing terminology is common in the primary literature. In this article, Endelman and Dawson advocate for standardized nomenclature and an alternative parameterization for diploid populations outside Hardy-Weinberg Equilibrium, using insights from the study of polyploids.

A key goal of quantitative genetics is the estimation of genetic effects from phenotypes, which are measured for a population in one or more environments. The concept of additive vs dominance effects dates to Fisher (1918) and remains relevant to current practice and research in genomic selection and genetic mapping. Despite this long history, these terms are used in confusing and imprecise ways in the scientific literature. In this article, we advocate for standardized nomenclature and an alternative parameterization for diploid populations outside Hardy–Weinberg equilibrium (HWE), using insights from the study of polyploids.

The additive effect of an allele is its average effect on the phenotype, from linear regression. For a single locus with *t* alleles in a population, the following model predicts the phenotype $y_{i}$ of individual *i* based on the dosage of its alleles, $X_{ij}\in \{0,1,2\}$ for allele *j*:


(1) $$y_{i}=\mu +\overset{t}{\underset{\;j=1}{\sum }}\;(X_{ij}-2p_{j})\alpha_{j}+\varepsilon_{i}$$

In Equation (1), allele dosages are “centered” by subtracting their population mean (two times allele frequency *p*), which makes intercept *μ* equal to the mean phenotype. The residuals include both dominance and nongenetic effects. Regression coefficient $\alpha_{j}\;$ is the **additive effect** of allele *j* and represents the average change in *y* when the dosage of allele *j* increases by 1, *holding all other variables constant.* This standard definition of a regression coefficient is biologically problematic, since increased dosage of one allele in an individual must be compensated by decreased dosage for another allele (without changing ploidy). This leads to the concept of an additive substitution effect, which can be defined for a locus with multiple alleles, but we now specialize to a biallelic locus with alleles 0 and 1 at frequencies *q* and *p*, respectively. By defining $X_{i}$ as the dosage of allele 1, $2-X_{i}$ is the dosage of allele 0, and Equation (1) becomes


(2) $$\begin{array}{rl} y_{i} & =\mu +(2-X_{i}-2q)\alpha_{0}+(X_{i}-2p)\alpha_{1}+\varepsilon_{i}\\ & =\mu +(X_{i}-2p)(\alpha_{1}-\alpha_{0})+\varepsilon_{i}=\mu +W_{A}\alpha +\varepsilon_{i} \end{array}$$

Equation (2) introduces the symbol $W_{A}$ as the additive regressor, which equals the centered dosage. By including only one allele dosage in the regressor, the regression coefficient changes from the additive effect of the allele in Equation (1) to the **additive substitution effect**, $\alpha =\alpha_{1}-\alpha_{0}$.

The multilocus generalization of Equation (2) is widely used for genomic prediction with biallelic markers, accompanied by slight changes in terminology and notation:


(3) $$\begin{array}{rl} y_{i} & =\mu +\underset{k}{\sum }\;W_{A,ik}\alpha_{k}+\varepsilon_{i}\\ W_{A,ik} & =X_{ik}-2p_{k} \end{array}$$

The symbol $\alpha_{k}$ now represents the additive substitution effect for locus *k*, not the additive effect of an allele, and the word “substitution” is often omitted. The **additive value** of an individual is the sum of its additive effects: $\sum_{k}W_{A,ik}\alpha_{k}$. Additive values are often called breeding values in the literature, although this is only strictly true under idealistic conditions of HWE without epistasis.

Confusion about additive effects can arise when dominance effects are explicitly modeled, rather than leaving them in the residual. Most readers will be familiar with the (*a*,*d*) parameterization of a single biallelic locus, in which 2*a* equals the difference between the homozygotes and *d* is the heterozygote deviation from the homozygote midpoint (Table 1). This has been called the **genotypic parameterization** (Vitezica et al. 2013) because the parameters are essentially the genotypic values (i.e. the expected phenotypic values). This model corresponds to regression on the indicator variable $\delta_{X,1}=X(2-X)$, which equals 1 for heterozygotes and 0 for homozygotes. The population mean of the indicator variable is the heterozygosity proportion, which can be represented as 2pq(1−f) for inbreeding coefficient *f* (Wright 1951). The centered form of the genotypic parameterization, which maintains the intercept as the mean phenotype, is thus


(4) $$\begin{array}{rl} y_{i} & =\mu +\underset{k}{\sum }\;W_{A,ik}a_{k}+\underset{k}{\sum }\;W_{d,ik}d_{k}+\varepsilon_{i}\\ W_{A,ik} & =X_{ik}-2p_{k}\\ W_{d,ik} & =X_{ik}(2-X_{ik})-2p_{k}q_{k}(1-f_{k}) \end{array}$$

Equation (4) introduces $W_{d}$ as the dominance regressor for the genotypic parameterization. Although the additive regressor is the same in Equations (4) and (3), the meaning of its regression coefficient changes because $W_{d}$ is not orthogonal to $W_{A}$ (the covariance is only zero when p=1/2). In Equation (3), the dominance effects are in the residual, which *is* orthogonal to $W_{A}$ from the properties of least-squares regression. The notation was changed from $\alpha_{k}$ to $a_{k}$ in Equation (4) because the regression coefficients are no longer additive (substitution) effects, and $\sum_{k}W_{A,ik}a_{k}$ is not the additive value. Some authors describe $a_{k}$ as “functional” or “biological” additive effects, but this is discouraged because it obscures the true meaning. The *a* parameter is unnamed in standard texts (Falconer and Mackay 1996; Lynch and Walsh 1998; Bernardo 2020), so we propose the name **homozygote parameter**. By analogy, *d* may be called the **heterozygote parameter**, although dominance parameter is also appropriate.

**Table 1.** Genetic description of a single, biallelic locus with inbreeding coefficient *f*.

Genotype	Allele dosage (*X*)	Genotype frequency	Genotypic parameterization	Additive value parameterization
00	0	$q^{2}+pqf$	$g_{00}=-a$	$g_{00}=\mu +2\alpha_{0}+\beta_{00}$
01	1	2pq−2pqf	$g_{01}=d$	$g_{01}=\mu +\alpha_{0}+\alpha_{1}+\beta_{01}$
11	2	$p^{2}+pqf$	$g_{11}=a$	$g_{11}=\mu +2\alpha_{1}+\beta_{11}$

Column 2 is the individual dosage of allele 1, with population frequency *p*. Genotypic values are denoted $g_{ij}$, with mean *μ*. Additive effects are denoted $\alpha_{i}$ and dominance effects $\beta_{ij}.$

To introduce dominance without changing the additive effects, the dominance regressor $W_{D}$ (with a capital D subscript) must have zero covariance with the additive regressor $W_{A}$. For loci in linkage equilibrium, Equation (5) is an orthogonal parameterization:


(5) $$y_{i}=\mu +\underset{k}{\sum }\;W_{A,ik}\alpha_{k}+\underset{k}{\sum }\;W_{D,ik}\beta_{k}+\varepsilon_{i}$$

The regression coefficient for $W_{D}$ is the **dominance substitution effect**, $\beta =\beta_{01}-\frac{1}{2}(\beta_{00}+\beta_{11}),$ which is a linear contrast of the dominance effects (Table 1). The Greek letter *β* is used for dominance effects to follow Greek letter *α* for additive effects. This is new terminology, which originated in polyploids but also applies to diploids (Wricke and Weber 1986; Endelman et al. 2018; Endelman 2023). In fact, the quantitative genetics of polyploids helps illuminate three distinct concepts in the theory of dominance—dominance deviation, dominance effect, and dominance value—which are often muddled in diploids because they collapse to the same quantity.

Consider the multiallelic, tetraploid generalization of the additive value parameterization (Kempthorne 1957), which uses standard notation from ANOVA and subscripts *ijkl* to represent the four homologous genes (at one locus) in an individual with genotypic value $g_{ijkl}:$


(6) $$\begin{array}{rl} g_{ijkl} & =\mu +\alpha_{i}+\alpha_{j}+\alpha_{k}+\alpha_{l}+\beta_{ij}+\beta_{ik}+\beta_{il}+\beta_{\;jk}+\beta_{\;jl}+\beta_{kl}\\ & \;+\gamma_{ijk}+\gamma_{ijl}+\gamma_{ikl}+\gamma_{\;jkl}+\delta_{ijkl} \end{array}$$

Analogous to diploids, the additive value is the sum of four additive effects (e.g. $\alpha_{i}$), one for each homolog. The **dominance deviation**, which is the residual from the additive effect regression, is composed of 6 digenic effects (e.g. $\beta_{ij}$), 4 trigenic effects (e.g. $\lambda_{ijk}$), and 1 quadrigenic effect $\delta_{ijkl}$, all of which are **dominance effects**. Dominance effect $\beta_{ij}$ has the same meaning in tetraploids and diploids: it is the average change in genotypic value, not explained by the additive effects, when the dosage of allele-pair *ij* increases by 1, holding other variables constant. A more biological interpretation is possible for the (digenic) dominance substitution effect, $\beta =\beta_{01}-\frac{1}{2}(\beta_{00}+\beta_{11})$, which is the average change in genotypic value when a heterozygous pair replaces a homozygous pair. Note that the dominance substitution involves no net allele substitution. Ironically, dominance effects in a polyploid are “additive” in the mathematical sense: from Equation (6), the digenic **dominance value** is the sum of 6 digenic dominance effects. For diploids, the dominance value is a single dominance effect (Table 1).

Returning to Equation (5), this has been called the breeding value parameterization at HWE (Vitezica et al. 2013), but we propose **additive value parameterization** as a more general name, since HWE is not required. The solution for $W_{D}$ at HWE can be found in standard texts (Falconer and Mackay 1996; Lynch and Walsh 1998; Bernardo 2020), and the most general solution requires two parameters to describe the deviation of genotype frequencies from HWE (Álvarez-Castro and Carlborg 2007; Vitezica et al. 2017). In many situations, however, HWE deviations can be described by one parameter, the inbreeding coefficient *f* (Table 1), which simplifies the result (Table 2 and Appendix). Whereas $W_{A}$ is a linear function of allele dosage, $W_{D}$ is a concave parabola (Fig. 1). The maximum occurs at *X* = 1 when *p* = 0.5 and shifts to lower (higher) values as *p* decreases (increases). Increasing *f* shifts the maximizer of $W_{D}$ back toward *X* = 1. Although the only biologically possible values of *X* are {0, 1, 2}, the quadratic formula is useful when fractional values are present in imputed marker data.

**Fig. 1.**
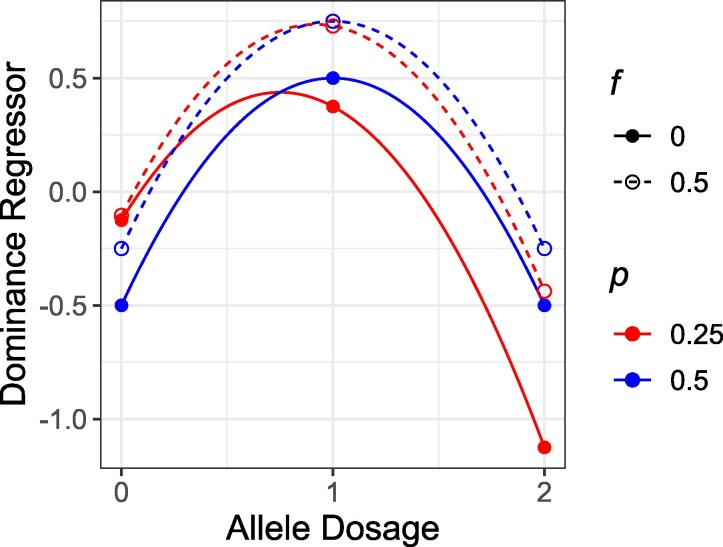
Quadratic relationship between allele dosage and dominance regressor *W*_D_, for different values of inbreeding (*f*) and allele frequency (*p*) in the population.

**Table 2.** Decomposition of genotypic value for a biallelic (0/1) locus with inbreeding coefficient *f*.

	Additive	Dominance
Individual value	$W_{A}\alpha =\{\begin{array}{c} 2\alpha_{0},\;\;\;\;\;X=0\\ \alpha_{0}+\alpha_{1},\;\;X=1\\ 2\alpha_{1},\;\;\;\;\;X=2 \end{array}$	$W_{D}\beta =\{\begin{array}{c} \beta_{00},\;\;\;X=0\\ \beta_{01},\;\;\;X=1\\ \beta_{11},\;\;\;X=2 \end{array}$
Regressor	$W_{A}=X-2p$	$W_{D}=-X^{2}+X\left[4pq+\frac{\;p^{2}(3+f)+q^{2}(1+3f)}{1+f}\right]-\frac{2p(1-f)(\;p+qf)}{1+f}$
Substitution Effect	$\alpha =\alpha_{1}-\alpha_{0}$	$\beta =\beta_{01}-\frac{1}{2}(\beta_{00}+\beta_{11})$
Effects	$\alpha_{0}=-p\alpha$ $\alpha_{1}=q\alpha$	$\beta_{00}=-2p^{2}\beta \left(\frac{1-f}{1+f}\right)\left(1+\frac{q}{p}f\right)$ $\beta_{01}=2pq\beta \frac{\left(1+\frac{q}{p}f\right)\left(1+\frac{\;p}{q}f\right)}{1+f}$ $\beta_{11}=-2q^{2}\beta \left(\frac{1-f}{1+f}\right)\left(1+\frac{\;p}{q}f\right)$

The individual dosage of allele 1 is *X*, with population frequency p=1−q.

The additive value parameterization is a useful model for genomic prediction, even though genome-wide markers are not in linkage equilibrium. In classical quantitative genetics, the genetic parameters are fixed effects, but the marker effects in genomic prediction are random (de los Campos et al. 2015). For genomic prediction by best linear unbiased prediction, the substitution effects are normally distributed, with zero mean for the additive effects, $\alpha_{k}∼N(0,\sigma_{\alpha }^{2}),$ but nonzero mean for the dominance effects, $\beta_{k}∼N(\mu_{\beta },\sigma_{\beta }^{2})$. Additive substitution effects have zero mean because the allele coding (0/1) is arbitrary, and therefore so is the sign (+/−). By contrast, the sign of the dominance substitution effect is unchanged under allele recoding and is related to inbreeding depression $I={\bar{g}}_{\mathrm{HWE}}-\bar{g}$ (Endelman 2023). For diploids, $I=\mu_{\beta }\sum_{k}2p_{k}q_{k}f_{k}$ represents the change in the population mean relative to a population in HWE with the same allele frequencies (Falconer and Mackay 1996).

In summary, we hope this short article will serve as a convenient reference on additive and dominance effects and promote proper use of quantitative genetics terminology. The genotypic (*a*,*d*) parameterization is perfectly valid for genetic modeling, but only the additive value (*α*, *β*) parameterization directly partitions genotypic value into additive and dominance components. This simplifies the analysis of variance (for unlinked loci), and selection on additive values promotes long-term genetic gain.

## Appendix: Deriving the additive value parameterization

From Equation (2), the regression problem for the additive substitution effect can be written as

(A1 )
$$z=\left[\begin{array}{c} g_{00}-\mu \\ g_{01}-\mu \\ g_{11}-\mu \end{array}\right]=\left[\begin{array}{c} -2p\\ q-p\\ 2q \end{array}\right]\alpha +\left[\begin{array}{c} \beta_{00}\\ \beta_{01}\\ \beta_{11} \end{array}\right]=w\alpha +e$$

where z is the vector of genotypic value deviations, **w** is the additive regressor, and e is the residual vector of dominance effects. The weighted least-squares solution is $\alpha =w^{\prime }Vz/w^{\prime }Vw$, where **V** is a 3 × 3 diagonal matrix of genotype frequencies. After extensive simplification, the result is

(A2 )
$$\alpha =a+d(q-p)\left(\frac{1-f}{1+f}\right)$$

Expressions for $\alpha_{0}$ and $\alpha_{1}$ (Table 2) follow from setting the population mean of the additive values equal to zero. The dominance effects are found by substituting Equation (A2) into Equation (A1), and when combined with the definition of the dominance substitution effect, the result is

(A3 )
β=d.
